# Paraoxonases Activities and Polymorphisms in Elderly and Old-Age Diseases: An Overview

**DOI:** 10.3390/antiox8050118

**Published:** 2019-05-02

**Authors:** Débora Levy, Cadiele Oliana Reichert, Sérgio Paulo Bydlowski

**Affiliations:** 1 Genetic and Molecular Hematology Laboratory (LIM31), Hospital das Clínicas, Faculdade de Medicina, Universidade de Sao Paulo, São Paulo 05419-000, SP, Brazil; d.levy@hc.fm.usp.br (D.L.); kadielli@hotmail.com (C.O.R.); 2 Center of Innovation and Translacional Medicine (CIMTRA), Department of Medicine, Faculdade de Medicina, Universidade de Sao Paulo, São Paulo 05419-000, SP, Brazil; 3 Instituto Nacional de Ciencia e Tecnologia em Medicina Regenerativa (INCT-Regenera), CNPq, Rio de Janeiro 21941-902, RJ, Brazil

**Keywords:** aging, paraoxonase, PON, lipids, lipid peroxidation, heart disease, atherosclerosis, diabetes, cancer, neurodegenerative diseases

## Abstract

Aging is defined as the accumulation of progressive organ dysfunction. There is much evidence linking the involvement of oxidative stress in the pathogenesis of aging. With increasing age, susceptibility to the development of diseases related to lipid peroxidation and tissue injury increases, due to chronic inflammatory processes, and production of reactive oxygen species (ROS) and free radicals. The paraoxonase (PON) gene family is composed of three members (*PON1*, *PON2*, *PON3*) that share considerable structural homology and are located adjacently on chromosome 7 in humans. The most studied member product is PON1, a protein associated with high-density lipoprotein with paraoxonase/esterase activity. Nevertheless, all the three proteins prevent oxidative stress. The major aim of this review is to highlight the importance of the role of PON enzymes in the aging process, and in the development of the main diseases present in the elderly: cardiovascular disease, diabetes mellitus, neurodegenerative diseases, and cancer.

## 1. Introduction

Over the years, several theories have emerged to better explain the aging process. Among them, the influence of oxidative stress on aging has received great attention. It is based on the fact that cell metabolism, even under physiological conditions, generates reactive bioproducts, free radicals and oxidizing metabolites. The unbalance between the generation of oxidative metabolites and the presence of antioxidants would be responsible for the initiation of the aging process. The oxidative damage generated by metabolites such as free radicals, Reactive Oxygen Species (ROS) and/or Reactive Nitrogen Species (RNS), and peroxides, compromises the structure and function of macromolecules (carbohydrates, lipids, proteins, and DNA) in both, the intracellular and extracellular microenvironments [1]. For example, polyunsaturated fatty acids peroxidation mediated by hydroxyl and peroxyl radicals produce highly reactive aldehydes such as *trans*-4-hydroxy-2-nonenal, malondialdehyde (MDA) and isoprostanes [2,3,4]. The amino groups as lysine and arginine react with the carbonyl groups of carbohydrates in a process called glycosylation, resulting in advanced glycation end products (AGEs). Major AGEs include hydroimidazolone, N∈-carboxymethyl-lysine, pentosidine, and glucosepane [2,3]. Oxidative damage to DNA results in several mutagenic lesions including 2-hydroxy adenine, 8-oxoadenine, 5-hydroxycytosine, cytosine glycol, thymine, and glycol. The mutagenic consequences of oxidative stress on DNA are 8-oxo-7,8-dihydro-guanine and 8-oxo-7,8-dihydro-2′deoxyguanosine lesions, which can result in G-to-T transversion events [2,3].

Oxidized lipids are able to trigger cell signaling in several cell types and tissues. Their biological effects are dependent on extent of lipid peroxidation and concentration, different transduction pathways, and nature of oxidized lipids (oxidized phospholipids, oxysterols, prostaglandins) [5,6,7,8,9,10,11,12,13]. One of the enzymes that inhibit lipid peroxidation is the paraoxonase family, composed by paraoxonase-1 (PON-1), paraoxonase-2 (PON-2) and paraoxonase-3 (PON-3). They are involved in several diseases in which lipid peroxidation is important, including chronic inflammatory processes caused by metabolic oxidative stress [14,15,16].

PON-1 is a key functional constituent of HDL particles; PON-2 is essentially found in mitochondria and endoplasmic reticulum while PON-3 is localized in HDL, mitochondria and endoplasmic reticulum [14,15,16]. Many studies have shown that the serum activity of PON-1 in individuals suffering from several diseases (ischemia, chronic kidney disease, morbid obesity, dyslipidemia), the esterase activity of the enzyme is considerably reduced. In addition, the administration of purified PON-1 itself was shown to reduce the development of inflammatory bowel disease and diabetes, both in animal model [17].

The elderly population is more susceptible to the oxidative damage generated by these molecules, due to the reduction of antioxidant molecules [18]. Older individuals have more difficulty in absorbing or producing certain nutrients, such as antioxidant vitamins (vitamin A and β-carotene, vitamin C and E) as well as polyphenolic compounds [19]. By genetic factors or environmental influence, there is a change in the detoxification and inactivation of toxic metabolites and xenobiotics by enzymes of the CYP450 complex and glutathione peroxidase [20]. Besides, with increasing age, a decrease in the activity of antioxidant enzymes such as catalase (CAT), superoxide dismutase (SOD) and paraoxonases (PONs), is observed [21]. The altered function of these enzymes leads to changes in gene expression, metabolic control and maintains cells in senescence [22]. Uncontrolled redox system promotes a vicious cycle with high and chronic inflammation and intense lipid peroxidation of membranes and tissues, thus decreasing the quality of life of the elderly population [23].

The diseases with a higher incidence and prevalence in the elderly population include type 2 diabetes mellitus, obesity, dyslipidemia, atherosclerosis, cardiovascular disease, acute myocardial infarction, hypertension, peripheral vascular disease, Alzheimer’s disease, Parkinson’s disease, age-related macular degeneration, multiple sclerosis, epilepsy, depression and cancer [24,25,26,27,28].

The aim of this paper is to review the pathophysiological role of paraoxonases: PON-1, PON-2 and PON-3, in the development of diseases related to aging and lipid peroxidation, mainly in atherosclerosis and cardiovascular diseases, diabetes mellitus, neurodegenerative diseases (Alzheimer’s disease, Parkinson disease, multiple sclerosis) and cancer.

## 2. The Paraoxonase Family

The three human *PON* genes are clustered on the long arm of chromosome 7 (7q21.31–q22.1). All of them have nine exons and share around 70% of homology at the nucleotide level and 60% of homology at the amino acid sequence [14,29]. In mammals, the comparative similarity of each of those three genes is higher and reaches 81–90% homology at the nucleotide sequence and 79–90% of homology at the amino acid sequence [29,30]. *PON-1* can be differentiated from *PON-2* and *PON-3* by the three extra nucleotide residues in exon 1 [29,30,31]. Although the three enzymes have high homology to each other, each of them has its particular function, according to its location. However, all paraoxonases act to protect against oxidative damage and decrease endoplasmic reticulum stress. They also participate in the regulation of apoptosis, in non-specific immunity, and in the bioactivation of drugs [28].

PON-1 was first described in 1946 when Abraham Mazur reported an enzyme activity found in mammalian tissues which was capable of hydrolyzing organophosphate compounds [32]. This led to the initial identification of the human serum paraoxonase (PON-1) enzyme in the early 1950s by Norman Aldridge. Initially, the enzyme was referred to as “A”-esterases, but later became known as paraoxonase due to its ability to detoxify the substrate paraoxon, the toxic metabolite of the commonly used insecticide parathion [32,33,34].

**Figure 1:**
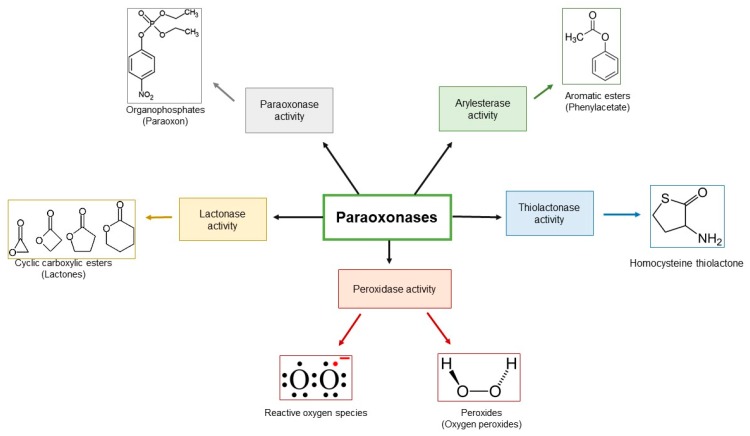
Paraoxonases activities and substrates.

The three paraoxonase (PON-1, PON-2 and PON-3) enzymes are able to, in vitro, hydrolyze aromatic lactones, as well as products derived from the enzymatic or non-enzymatic metabolism of both, arachidonic and docosahexaenoic acid [35,36,37,38]. However, although all three PON types are named paraoxonase, only PON-1 has the ability to hydrolyze organophosphates [35]. The PON-1 can hydrolyze toxic oxidative metabolites of organophosphorus insecticides (parathion, diazinone and chlorpyrifos), nerve agents (sarin and soman), aromatic esters, and aliphatic lactones. The PON-2 has the ability to hydrolyze and inactivate N-acyl-homoserine lactones, which are quorum signs of pathogenic bacteria. The PON-3 hydrolyzes drug metabolites and is involved in the multidrug resistance phenotype [36,37,38] (Figure 1).

### 2.1. Paraoxonase-1

PON-1 is a calcium-dependent enzyme consisting of 354 amino acids with a molecular mass of about 43 kDa, synthesized in the liver [34]. Structural analysis using X-ray crystallography revealed the three-dimensional structure of PON-1. It is composed of six-bladed β-propeller, forming a central tunnel which contains two calcium ions (Ca^2+^) [39]. Each calcium ion, depending on its location within the enzyme, plays an important role in the activity of PON-1. The calcium ion located more deeply within the tunnel has a structural role and maintains the conformational stability of the enzyme [39]. The other calcium ion at the bottom of the active site cavity has a catalytic role and is important for substrate positioning and activation of the ester bond [39]. Three helices are located above the active site of PON-1, named H1, H2 and H3, where H1 and H2 have functions in the PON-1-HDL interactions [34,39].

**Figure 2:**
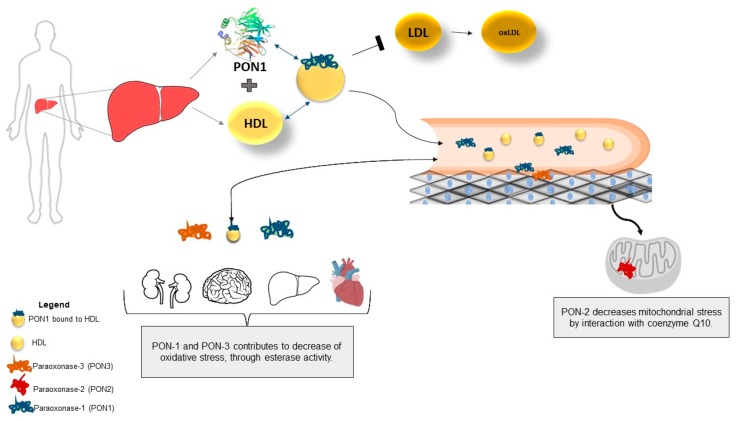
Location and distribution of PON-1, PON-2 e PON-3 in organs and tissues.

PON-1 has paraoxonase, arylesterase, and lactonase activities, being one of the important enzymes associated with high density lipoproteins (HDL) [40]. In fact, PON-1 is one of the enzymes responsible for the antioxidative activity of HDL, that includes lecithin-cholesterol acyltransferase and platelet-activation factor acetyl-hydrolase [40]. Although in general PON-1 circulates associated with HDL, it is also found, in a lesser extent, in very low density lipoproteins (VLDL) and chylomicrons in the postpandrial period [41,42]. The stable and high affinity binding between PON-1 and HDL requires the interaction with apoA-I [43]. The high lipolactonase activity of PON-1 is stimulated by apoA-I, and free PON-1 has lactonase activity lower than PON-1 associated with HDL [43,44]. HDL-bound PON-1 is transported and distributed to the cell membranes, in several tissues and organs (Figure 2). In this way, it exerts a primordial role against lipid peroxidation of membranes and low-density lipoprotein (LDL) [34,45], as well as the generation of free radicals resulting from the activation of inflammatory processes and oxidative stress [45].

The enzymatic activity of PON-1 has several interferences, and the micro- and macro-environment may contribute to reduce the activity of the enzyme, as well as, a mutation and/or presence of single-nucleotide polymorphism (SNP) in the promoter and/or codon region of the *PON-1* gene [46]. Currently, the enzyme is known to have lower serum arylesterase activity in individuals with higher oxidative stress, it is a protective enzyme against both tissue and lipid damage caused by oxidative stress and its bioactive derivatives [47,48,49,50,51,52,53]. PON-1 has peroxidase activity, leading to the neutralization of hydroperoxides of fatty acids, cholesteryl ester hydroperoxides and hydrogen peroxide (H_2_O_2_) [54]. The high expression of PON-1 contributes to the hepatoprotection against the peroxides generated in the inflammatory process [55].

PON-1 also acts in the hydrolysis of homocysteine thiolactone (HTL), a product of homocysteine (Hcy) metabolism and a poor substrate for PON-1. Homocysteine is part of the sulfur amino acid group, and its formation occurs by the demethylation of methionine obtained from diet or the catabolism of homocysteine itself [56,57]. Hcy-thiolactone is a reaction product catalyzed by the enzyme methionyl tRNA synthetase. Hcy-thiolactone is a reactive metabolite that modifies protein lysine residues in a process known as N-homocysteinylation. Plasma Hcy-thiolactone and the N-homocysteinylation protein are present in the human body and are greatly elevated in hyperhomocysteinemia of genetic or nutritional origin [57].

It was hypothesized that vascular disease mediated by Hcy is due to its toxicity by its conversion to Hcy-thiolactone, which then modifies the proteins to generate N-Hcy-protein, leading to cell death, altered vessel structure, chronic inflammation, autoimmune response and atherosclerosis, as well as the development of thrombosis and neurological diseases [58,59,60]. N-Homocysteinylation of HDL changes its structure, the conformation and properties of apolipoprotein, decreasing its antioxidant properties [58]. In vivo and in vitro studies demonstrate that PON-1 protects against the damage caused by hyperhomocysteinemia associated with the development of atherosclerosis and vascular endothelial dysfunction [61], age-related macular degeneration [62], multiple sclerosis [63] and cancer [28]. In an animal model, PON-1 has been shown to be protective against the neurotoxicity of Hcy-thiolactone by hydrolyzing it in the brain [64].

#### PON-1 Polymorphisms

Eight SNPs have been identified on the promoter region and 176 within the human *PON-1* gene sequence. Among the SNPs described for *PON-1* gene, two gained special attention due to the association between paraoxonase, arylesterase and lactonase activities of the different alloenzymes [39]. The arginine aminoacid at position 192 of PON-1(Q192R; gln191 to arg) specifies high-activity plasma paraoxonase, whereas glutamine at this position specifies a low-activity variant [65]. The glutamine determines the A allozyme and the arginine amino acid determines B allozymes of PON-1 [66]. The exchange of a leucine at position 55 by a methionine (polymorphism L55M) increases paraoxon activity over 50% in the homozygous form (MM) compared with the LL and LM [67]. Moreover, 55L maintains stability of the PON-1 protein structure [68]. In addition, frequencies of Q192R and L55M alleles are different between populations. The frequency of alleles Q192 and L55 is higher in Caucasian populations, while in Asian populations the allele R192 is higher, and the allele M55 is lower [28]. Moreover, the enzymatic activity of PON-1 varies according to the populations, as well as the susceptibility to develop certain diseases related to paraoxonases [28]. The HTLase activity of PON-1 is modified by polymorphisms at position 55 and 192. Individuals carrying the alleles both L55 and R192 have higher homocysteine lactonase activity than individuals carrying PON-1 M55 and Q192 alleles [58]. Other three SNPs (G-907C, A-162G and C-108T) in the promoter sequence of the *PON-1* gene were associated with considerable differences in PON-1 concentration and activity [39].

### 2.2. Paraoxonase-2

The human *PON-2* gene is located on the long arm of chromosome 7 (7q21.3–q22.1) and is adjacent to the family members *PON-1* and *PON-3*. The genomic structure of *PON-2* is composed of nine exons that encode a protein of 355 amino acids, with approximately 40–43 kDa; such structure presents 65% similarity with PON-1. The difference in the mass of PON-2 is due to the presence of an additional glucose residue (43 kDa) in the structure of the enzyme. However, there is no difference in enzyme function [69]. PON-2 is an intracellular protein and, unlike PON-1 or PON-3, it is not present in plasma. Based on a phylogenetic analysis, PON-2 is the oldest member of this gene family, and PON-1 and PON-3 have evolved from PON-2 [29,70].

PON-2 is located in the membrane of the mitochondria and in the endoplasmic reticulum. Due to its location, expression and lactonase activity in all tissues, it has gained special attention in studies related to oxidative stress and lipid peroxidation. Its expression is upregulated under cellular oxidative stress and reduced superoxide release from the inner mitochondrial membrane. By its anti-oxidative effect, PON-2 reduces cellular oxidative damage and influences redox signaling, which promotes cell survival and decrease of apoptosis [71,72,73]. PON-2 mRNA is expressed in almost all human tissues, with the highest expression in the liver, lungs, placenta, testicles and heart [74]. PON-2 mRNA is also found in the cells of the arterial wall, including endothelial cells, smooth muscle cells and macrophages [74,75,76]. Among the three paraoxonase enzymes, PON-2 is the only one expressed in nervous tissues [69]. Studies in mice have shown that both PON-2 concentration, mRNA expression and lactate activity of the enzyme are different between males and females. The high concentration of PON-2 in brain (in females, about three times higher than in males), protects neurons and astrocytes against oxidative stress toxicity and lipid peroxidation [77]. Furthermore, the antioxidant, anti-inflammatory and protective effect of PON-2 has been shown in intestinal epithelial cells [78,79], in human vascular endothelial cells, lung epithelial carcinoma cells and in Caco-2/15 intestinal epithelial cells [78,79,80]. In vitro studies suggest that PON-2 can regulate the activity of epithelial sodium (Na^+^) channels by modulating its intracellular traffic and surface expression. Epithelial sodium channels are involved in physiological processes such as mechanosensitivity, locomotion, nociception, termination of seizures, detection of pheromones, release of fluid in the airways, among others [81].

*PON-2 polymorphisms.* The most important and studied polymorphisms of the *PON-2* gene are an Ala/Gly substitution at position 148, and a Ser/Cys substitution at position 311 [82].

### 2.3. Paraoxonase-3

The *PON-3* gene was described in 1996 by Primo-Parmo et al., [29], and has about 85% of similarity with *PON-1* gene. PON-3 is a 40-kDa glycoprotein mainly synthesized by the liver. Like PON-1, PON-3 is found in circulation tightly bound to HDLs. PON-3 has high lactonase activity, low arylesterase activity, and no paraoxonase activity [16,83,84]. The activity of PON-3 has been reported to be calcium-dependent, like PON-1. PON-3 hydrolyzes aromatic lactones and lactones composed of five and/or six membered rings with aliphatic substituent groups, such as statin prodrugs. However, simple lactones or lactones with polar substituent groups are not hydrolyzed by PON-3 [16]. PON-3 expression has been described in endoplasmic reticulum of intestinal cells and more recently in mitochondria of selected tissues [33]. During oxidative stress in mouse macrophage PON-3 expression decreases, while PON-2 expression increases, highlighting a stronger antioxidant role of PON-2 in relation to PON-3 [84]. Interestingly, in vitro, PON-3 was described to protect LDL from copper-induced oxidation [16].

*PON-3 polymorphism.* There are very few studies on polymorphisms of the *PON-3* gene. The main polymorphisms in *PON-3* described so far are the c.449G > A, the SNPs A10340C and A2115T, and the PON-3 intronic variant INS2+3651 (A > G) (rs10487132) [83].

## 3. Paraoxonases Association with Diseases

Paraoxonases activities and polymorphisms have been studied and often associated with several different diseases: chronic metabolic syndrome, chronic liver impairment, psoriasis, systemic lupus erythematosus, hearing loss, sporadic amyotrophic lateral sclerosis and obesity. It seems that PON also have a role in infectious diseases. Infectious diseases are often associated with oxidative stress and an inflammatory response [85,86,87].

The acute-phase response (APR) to an infection generates metabolites that alter the composition and structure of HDL: depletion of cholesterol esters and an enrichment in free cholesterol, triglycerides, and free fatty acids. Overall, these changes cause HDL particles to lose some of their anti-atherogenic and anti-inflammatory properties and may even become pro-atherogenic and pro-inflammatory [88]. Thus, constant infections can lead to the development of heart disease. For instance, *Helicobacter pylori* infection is considered to be a risk factor for atherosclerosis. During the infection, increase in the levels of lipid hydroperoxides and PON-1 esterase activity is reduced [89,90].

Another example is the HIV-1 infection. It has been shown that PON-1 arylesterase activity correlates positively with the total number of CD4+ T cells; moreover, in individuals with high viral load PON-1 activity is reduced, while after reduction of viral load per treatment, PON-1 activity increases [51]. In addition, PON-3 concentrations increased about three times in HIV-infected patients with oxidative stress when compared with healthy controls. However, no significant association was seen between serum PON-3 concentrations and CD4+ T-cell, CD8+ T-cell, the CD4+/CD8+ ratio, and the plasma HIV-1 viral load [91].

## 4. Paraoxonases and Aging

Serum PON-1 concentration and activity are very variable between individuals. The enzymatic serum activity of PON-1 in healthy humans is low in early life, increases over time, remains stable throughout adult life, and decreases in the elderly [13,65].

During the process of aging, the equilibrium between redox state and antioxidant agents changes [92]. These alterations interfere with cellular and tissue regeneration [93]. The imbalance in the redox state may be associated with genetic and/or epigenetic alterations with increase of pro-inflammatory cytokines, failure to capture and use glucose as energy source, and increased oxidation of fatty acids and lipids [94,95]. Changes related to oxidative lipid metabolism are one of the main causes of the development of chronic diseases in the elderly as well as the decrease in longevity [96]. These changes may be due to genetic mutations, a sedentary lifestyle, or an unbalanced diet (rich in fats, sugars and carbohydrates), Inflammation, change in liver and renal functions, factors related to social interaction and the environment [97]. Among the protective factors against aging, the antioxidant enzymes are of importance, as previously described.

The association between PON-1 enzyme and cell aging was evidenced by Lee et al., [98]. PON-1 gene expression of human microvascular endothelial cells in culture was inhibited with interfering RNA. It was observed that cell viability was reduced, and cells entered into senescence, with increased β-galactosidase and flattened cellular morphology. Another important result was the high increase of protein hydroperoxides. Protein hydroperoxides inhibit thiol-dependent cysteine proteases and protein tyrosine phosphases [98]. These proteases break down proteins damaged by oxidative stress in cells, tissues and plasma, and participate in the activation and propagation in signaling pathways of the redox system. Therefore, when these proteases are decreased, the oxidative signaling is increased, contributing to the triggering of the aging process [98].

In a study with 1,932 Danish individuals, aged 47 to 93 years, three polymorphisms of the PON-1 enzyme, L55M, R192Q, T-107C, were investigated and related to mortality [99]. It was concluded that there was no difference between the genotypic and haplotypic distribution among the age groups evaluated. However, individuals with homozygous genotype for 192RR had a lower survival rate compared to 192QQ homozygous individuals, more pronounced in women. However, the meta-analysis conducted by Caliebe et al., [100], composed of 9580 individuals in different populations did not demonstrate an association between the R allele and longevity; however, the authors pointed out that due to the influence of different genetic and/or environmental factors, it is unlikely that the phenotypic effects of the genes are identical in different populations.

Another polymorphism of PON-1, the SNP rs662 (G > A), was associated with human longevity and decreased serum lipid concentration [101]. The alteration of lipid profile, increase of triglycerides and LDL-cholesterol are factors that decrease longevity and are predictors of many diseases related to lipid oxidation and increase of oxLDL [101]. In a study with 446 healthy older-old ‘Super-Seniors’ (individuals 85 or older who have never been diagnosed with cancer, cardiovascular disease, dementia, diabetes or major pulmonary disease), the genes that “protect” against aging and are related to the lipid profile were evaluated [102]. It was found that Super-Seniors did not carry an APOEε4 allele or an HP2 allele of haptoglobin, and that the lipid profile and cholesterol levels remained stable, as PON-1 and apoE. ApoE, as PON-1, play an important role in lipid metabolism and protection of lipid peroxidation [102]. Moreover, a synergism was found between the APOEε4 allele and the PON-1 L55M SNP, since individuals carrying both the APOEε4 allele and the 55MM homozygote genotype had a decrease of the arylesterase activity [102].

Although several studies did not associate the esterase activity to longevity, it was shown in individuals aged 20 to 81 years of both genders that a decrease in the arylesterase activity of PON-1 was proportional to the increase in the susceptibility of oxidation of the LDL (oxLDL) due to the increase of free radicals in the plasma. PON-1 was categorized as a longevity gene [103].

In the aging process, metabolic rates decrease; DNA is exposed to free radicals, peroxides and reactive oxygen species; the DNA methylation process and acetylation of chromatin increase with age, and as a consequence, there is a significant reduction of protein synthesis and accumulation of progressive organic dysfunction [104]. Sirtuins (STR) have become famous molecules able to retard aging and decrease age-related disorders [105]. They are located in several cell compartments: nucleus, cytoplasm and mitochondria [106]. These proteins protect cells against oxidative stress, regulate glucose/lipid metabolism, and promote the deacetylation stability of various substrates by regulating cell cycle processes, proliferation, and apoptosis [107]. In H_2_O_2_-producing cells under oxidative stress, mitochondrial PON-2 and STR2 proteins are overexpressed; this leads to a decrease in the damage caused by the peroxides, maintaining the mitochondria homeostasis [108]. In THP-1 monocytic cell line incubated with HDL isolated from healthy individuals, SIRT1 protein expression is increased compared with cells incubated with HDL from patients with coronary disease. Moreover, HDL from coronary disease patients has decreased PON-1 activity compared with that from healthy controls [109]. Interestingly, elderly individuals bearing mutant allele (G) for both rs7895833 (A > G) and rs7069102 (C > G), polymorphisms encoding the STR1 protein, had lower levels of PON-1 [110].

## 5. Paraoxonase and Cardiovascular Diseases

Aging is a risk factor for atherosclerosis. Atherogenesis is a slow and progressive process that begins in the first decades of life until complete plaque formation at the end of adulthood/onset of aging [111]. The process is multifactorial and includes alterations, dysfunction and activation of endothelial cells, smooth muscle cells and macrophages in the arterial wall, beside lipoproteins and humoral factors. Inflammatory cells and inflammatory mediators are also present at all stages of plaque formation [112]. The arterial permeability is increased, reactive oxygen species are generated, and inflammatory adhesion proteins and chemokines are expressed. The endothelium permeability allows an influx of plasma components, in the subendothelial area, where lipoproteins undergo several changes, including oxidation [113]. Chemokines and adhesion proteins promote the recruitment of monocytes that take up modified lipoproteins, accumulate lipids (mainly cholesterol esters) and are converted into macrophagic foam cells that form fatty streaks. These early lesions may rapidly grow in the presence of hypercholesterolemia or other risk factors, leading to the fibrous plaque [114,115,116].

The development of atherosclerosis also involves the oxidation of LDL, producing a pro-inflammatory bioactive particle necessary for the onset and formation of atheroma plaque [115,116,117]. With aging, there is an increase in ROS production from disruption of endothelial cells and tissues favoring oxidation, glycosylation, carbamylation and glycoxidation of plasma LDL [118]. On the other hand, HDL has been considered atheroprotective, due to its participation in the reverse cholesterol transport, where ApoA-1, the major apoprotein forming HDL, plays an important role by promoting the cholesterol efflux from macrophages. HDL is also known by its capacity to impair LDL oxidation, as stated before [118,119,120].

Descriptions relating paraoxonases to cardiovascular diseases began around 30 years ago with the association of decreased PON-1 activity with the development of atherosclerotic plaque [121]. Overexpression of PON-1 in mice increases approximately 30% the ability of HDL to mediate cholesterol efflux of J774 macrophages. It also caused an increase in the ability of macrophages to transfer cholesterol to ApoA-1, as well as increased both mRNA and ABCA1 protein expression, PPARγ and LXRα pathway [122].

However, PON-1 is not the only paraoxonase enzyme involved in the transport of cholesterol by macrophages. High PON-2 and PON-3 expression favor macrophage cholesterol efflux and impair oxidation of LDL by lipid hydroperoxide hydrolysis and increase the antioxidant action of HDL in protecting LDL [123,124]. As stated before, HDL is described as having the antioxidant ability to prevent or delay the oxidation of LDL due to proteins associated with its surface, such as PON-1, which can prevent the oxidation of HDL, as well as LDL [125,126,127,128,129,130,131,132,133,134,135,136,137]. Again, it is important to note that both PON-1 and PON-3 are components of HDL and that they reduce the atherogenicity of lipoproteins through of hydrolyzing of lipolactones [138,139]. As a result, the uptake of atherogenic lipoproteins by macrophages and the formation of foam cells is reduced [140]. The action of PON-1 and PON-3 can occur in both the vascular and plasma membranes, increasing the action spectrum of the enzyme [141,142]. It has been speculated that the mechanism by which PON-1 delays LDL oxidation involves the hydrolysis of the truncated fatty acid phospholipid hydroperoxides, cholesterol esters, and triglycerides, resulting in the production of lysophospholipids, cholesterol, diglycerides and oxidized fatty acids. However, these mechanisms have not yet been fully described [143].

The atheroprotective capacity of paraoxonase enzymes has been also attributed to the neutralization of compounds generated by mitochondria. The enzymes PON-2 and PON-3 interact with mitochondria coenzyme Q10, leading to decreased oxidative stress [143,144]. As a result, ROS-triggered mitochondrial apoptosis and cell death are reduced, decreasing atherosclerotic plaque development [143,144].

In PON-3 knockout mice, PON-3 deficiency resulted in compromised mitochondrial respiration due to decreased activity of complexes II, III and IV, which led to increased mitochondrial superoxide levels and increased ROS production. Another interesting result of this study was the increase in the expression of Mcp-1 and interleukin-6 (IL-6) and increase in calcified atherosclerotic plaque by up to 60% [144].

The altered relationship between PON-2 and mitochondria can also be observed in acute myocardial infarction (AMI); the lack of oxygen and the increase in ROS production are the main factors for cardiac tissue necrosis. The production of reactive oxygen species in the mitochondria, due to ATP depletion, causes the death of cardiomyocytes, following myocardial ischemia-reperfusion injury (IRI) [145]. In an animal model, PON-2 protects against acute myocardial infarction by reducing mitochondrial dysfunction and oxidative stress in cardiomyocytes by the PI3K/Akt/GSK-3β pathway [146]. As previously mentioned, PON-2 is located on the inner membrane of mitochondria, and PON-2 deficiency alters mitochondrial function by decreasing both mitochondrial complex I-III activity and ATP levels. Next, mitochondrial oxidative stress increases after increasing mitochondrial superoxide production and lipid peroxidation and decreasing reduced glutathione levels [146].

In addition, it has been shown that inflammation, reactive oxygen species and lipid peroxidation products generated during oxidative stress could enhance tissue factor (TF) expression and procoagulant activity. TF is the initiator of the extrinsic coagulation pathway [147]. In an experiment with PON-2 (−/−) mice, in order to evaluate the relationship between PON-2 and coagulation, Ebert et al., [148], observed an increase in oxidative stress, endothelial dysfunction and the concentration of interleukin-6 in the vasculature, as well as increased tissue factor activity in endothelial cells, *in vitro*. In this experiment it was also possible to verify that coagulation times were shortened and platelet procoagulant activity increased in PON-2 (−/−) mice. The reexpression of PON-2 in endothelial cells recovered the procoagulant state of rats. The authors concluded that the regulation of the redox state by the enzyme PON-2 regulates the activity of the tissue factor, preventing both activation of systemic coagulation and inflammation [148].

PON-1 polymorphisms seem also to affect the atherosclerotic process and cardiovascular disease. The G allele (rs662) in PON-1 gene was shown to be a risk factor for the development of arterial coronary disease in the Chinese population [149]. The GG and AG genotype and G allele of PON-2 (148 A/G) was associated with decrease in PON-1 activity and decrease of coronary heart disease in a Chinese population [150]. A study of 442 elderly patients with acute myocardial infarction followed up for a period of one year showed that patients with the C allele (S311C) of PON-2 gene had a worse prognosis when compared to patients without the C allele; that mortality risk was approximately 11 times higher [151]. It is important to note that this C311 polymorphism has been also associated with the development of coronary artery disease [152].

## 6. Paraoxonases and Type 2 Diabetes Mellitus

Oxidative stress and lipid peroxidation have been implicated as contributors to both the onset and progression of diabetes and its complications. The main consequences of long-term oxidative damage are the development of β-cell dysfunction, impaired glucose tolerance and mitochondrial dysfunction. By-products from oxidative stress activate several stress pathways such as NF-κB, JNK/SAPK and p38 MAP, which alter insulin signaling, intracellular and between cells, as well as contribute to peripheral insulin resistance in adipose and muscular tissues [153]. As mentioned previously, the paraoxonases exert various functions in order to reduce the oxidative environments.

In a study from Koren-Gluzer et al., [154], it was observed that PON-1-deficient mice (−/−), being fed a hypercaloric diet, develop peripheral resistance to insulin. The authors attributed this result to increased oxidative stress, increased p38MAPK activity, and decreased insulin-mediated tyrosine phosphorylation of insulin receptor substrate 1 (IRS-1); thereafter, there was a decrease in glucose uptake. After the addition of PON-1 to cultured myocytes *in vitro*, there was an increase in mRNA and protein expression of the glucose transporter GLUT-4. This increase was time- and PON-1 concentration-dependent. In addition, intracellular glycogen stores increased. These effects were mediated via the inhibition of the p38 MAPK pathway, which contributed to the reduction of IRS-1 serine phosphorylation and IRS-1 tyrosine phosphorylation. Another interesting result described was the blocking of the SH group in PON-1, which partially decreased GLUT4 expression. However, a mutation in H115 abolished GLUT4 expression [154]. The H115 residue forms part of the catalytic center of the enzyme PON-1, along with two more amino acid residues, L69, V346. Together, these residues are responsible for the hydrolase activity of PON-1 [155]. In addition, in vitro, PON-1 protects β-cells against cytokine-induced cytotoxicity (IL-1β, TNF-α and INF-γ), decreasing EROS, nitrous oxide levels and caspase-3 [156].

Changes in PON-2 have also been shown to influence the development of diabetes. In mice lacking the *PON-2* gene showed to have a greater probability of gaining weight, developing obesity, as well as hypertrophy of adipocytes, glucose tolerance in fasting, decreased oxygen consumption, energy expenditure and mitochondrial dysfunction [157]. In patients with T2D, the daily intake of 2 g of eicosapentaenoic acid (EPA) was shown to be efficient for PON-2 expression increase and decrease of glycemic indexes [158].

Obesity, lipid oxidation and its comorbidities are associated with impaired cognitive performance, accelerated cognitive decline, and neurodegenerative pathologies, such as dementia in old age, and interventions targeting obesity in middle age may be beneficial in reducing cognitive risks associated with age [159]. Blood PON-1 arylesterase, paraoxonase, and lactonase activities were evaluated in patients with morbid obesity before and six months after Roux-en-Y Gastric Bypass (RYGB). Before surgery, patients had a lower concentration of PON-1, with no change in arylesterase and paraoxonase activities, and higher lactonase activity, compared with non-obese individuals. Lactonase activity decreased after surgery, which was associated with increased esterified cholesterol levels. However, no increase in both arylesterase and paraoxonase activities were observed after surgery [160,161].

Genotyping of PON-1 Q192R and L55M polymorphisms was performed in 287 patients with type 2 diabetes and 293 healthy individuals. The frequency of the QR/RR and LM/MM genotypes was significantly higher in patients with type 2 diabetes compared to healthy individuals. In addition, subjects with the R allele had an increased risk to develop diabetes up to 1.68 times, and individuals with an M allele had a 2.24-fold risk of developing diabetes. The QR/RR and LM/MM genotypes were associated with higher body mass index (BMI), as well as increased cholesterol, triglycerides, LDL and fasting serum insulin and HOMA-IR [162].

The PON-2 gene polymorphisms 148 A/G and S311C have been independently associated with diabetic nephropathy in Type II diabetic patients. The susceptibility to diabetic nephropathy was intensified by the degree of Obesity [163].

A study conducted on 40 Iranian patients with type 2 diabetes, aged 40–65 years, associated the A allele (SNP PON-2-G148A), and C allele (SNP PON-2-C311S) with quantitative insulin sensitivity check index (QUICKI) and homeostasis model assessment for β-cell function (HOMA-BCF). They observed a relationship between PON-2 and insulin resistance [164]. However, patients sharing the 55 M allele (PON-1 L55M) were prone to having good glycemic control, independent of the presence of PON-2-G148A and PON-2-C311S [165]. Furthermore, the allele T of the polymorphism (−108C > T) of PON-1 gene was associated with decreased paraoxonase activity in type 2 diabetic patients; moreover, the frequency of the homozygous TT and heterozygous TC genotype were higher in these patients [166].

## 7. Paraoxonase and Neurodegenerative Diseases

PON-1 and its ability to hydrolyze organophosphates were the first described associations between paraoxonase and the development of neurodegenerative diseases. Lipid peroxidation in the nervous system compromises the myelin sheet, most of times being an irreversible process [167]. Decreased PON-1 activity and decreased PON-2 expression were associated with nervous system oxidative stress and the development of dementias [25].

Alzheimer’s disease (AD) is the most common cause of dementia among the elderly over the age of 65 years. As a neurodegenerative disorder, it is characterized by intracellular neurofibrillary tangles and extracellular senile plaques which consist of amyloid β-peptide (Aβ). Oxidative stress and lipid peroxidation are some of the causes of neurodegenerative disease development [168]. It has been suggested that paraoxonase activity is significantly lower in AD patients. In fact, the presence of PON-2 in the brain confers a protective effect against oxidative stress and neuroinflammation in brain cells, through its ability to eliminate reactive oxygen species (ROS) by exposure to oxidants [33]. Polymorphic changes in several genes such as Cryptochrome Circadian Regulator 1 (CRYL1), ApoE and haptoglobin (HP) have been described in patients with Alzheimer’s disease. They were associated with dysregulation of lipid metabolism and cholesterol, as well as with decreased life expectancy [169]. As described above, it is well known that individuals carrying PON-1 R allele have a higher PON serum activity as those carrying the Q allele. Thus, the involvement of this SNP in patients with AD is not surprising and may be related to its differential activity as an antioxidant enzyme [170]. Another SNP involved in the decrease of PON-1 arylesterase activity in patients with AD is −108C > T. in which the lowest enzymatic activities were observed in carriers of the CT and TT genotypes in both sexes [171]. The allele C, of the PON-2 S311C polymorphism, was considered one of the risk factors that can contribute to the development of Alzheimer’s disease in the Chinese population [83].

Parkinson’s disease (PD) is a disease in which the onset of symptoms occurs in the middle or advanced ages. Clinically, the disease is characterized by resting tremor, stiffness, akinesia and postural disorder. In the disease, death of dopaminergic neurons in the compact zone of the substantia nigra and the appearance of Lewy bodies in the neuronal cytoplasm are observed [172]. However, the etiology of the disease is unknown, although some theories involve prolonged exposure to toxic chemicals, such as organophosphate pesticides (OP) [170]. Membrane lipid peroxidation products have been also associated with the etiology of PD [170]. The involvement of PON-1 in the disease is related to the unbalance in dopamine concentration of acetylcholine (Ach), whose effects are opposed to those of dopamine in the striatum. Exposure to organophosphorus increases the levels of Ach due to blocked cholinesterase (ChE) by non-degraded organophosphates, which in turn induces overproduction ChE as feedback response [25]. In a study with 246 patients with PD exposed to organophosphates, the L55M polymorphism was described to influence some symptoms of PD progression [173]. 55MM homozygous genotype was associated with a faster progression of motor symptoms, the development of depressive symptoms, and a decrease in the cognitive ability in these patients [173]. This is interesting because the 55MM genotype has been associated with lower PON-1 esterase activity, and several studies have associated lower PON-1 activity with an increased risk of both depression and dementia, and decreased cognition in elderly [174,175,176].

Another neurodegenerative disease associated with PONs is multiple sclerosis (MS). Multiple sclerosis is a neurological, chronic and autoimmune disease. An oxidative stress promoted by a respiratory burst of invading and resident macrophages and astrocytes triggers a chronic and irreversible inflammatory process, and leads to demyelination, axonal damage and degeneration [177]. During the inflammatory process, a decrease of antioxidants is observed; moreover, PON-1 paraoxonase and arylesterase activities are decreased [178]. Castellazzi et al., [179] have associated the decrease of PON-1 arylesterase activity with the development of dementia in MS, due to redox state dysregulation and increased lipid peroxidation. The SPNs PON-1 L55M and R192Q, however, seem to have no influence on paraoxonase and arylesterase activities [180]. Thus, the reduction of PON-1 esterase activity in multiple sclerosis is probably a characteristic of the disease itself, since the presence of these polymorphisms does not confer a risk factor for the development of MS [181].

Gulf War (GW) syndrome is a disorder that appear to have affected many, but not all, Gulf War veterans, who came in contact with organophosphates compounds. Fatigue, widespread pain, cognitive and memory problems, skin rashes, gastrointestinal and respiratory difficulties were commonly reported. Therefore, not every veteran afflicted by GW illness show identical symptoms. Neurological problems include amyotrophic lateral sclerosis, brain cancer, seizures, neuritis and neuralgia, migraine headaches, multiple sclerosis and Parkinson’s disease [182]. As organophosphate pesticides intoxication is implicated in this disease, PON-1 activity and polymorphisms have been studied in GW veterans. PON-1 activity was shown to be reduced in patients [131,183]. R allele (heterozygous QR or homozygous RR) was prevalent in patients with neurological symptoms [184].

## 8. Paraoxonases and Cancer

The intense oxidative stress and inflammation process, in the long term, can lead to the carcinogenesis, besides maintaining an ideal environment for the growth of a tumor cell. Cancer cells produce high concentrations of ROS that alter cellular metabolic pathways and mitochondrial and peroxisome functions, and increase cell receptor signaling, inflammatory cytokines and activation of oncogenes [28].

Lower serum PON1 activity has been reported in cancer patients [185]. The signal transduction pathways involved in the modulation of PON1 are PKC, p44/42 and MAPK/ERK, and these pathways are activated by growth factors and other effectors to regulate cell growth and differentiation, apoptosis and angiogenesis [28]. Moreover, in individuals who have been treated for cancer, oxidative stress markers decreased, and the activities of the enzymes antioxants, superoxide dismutase, catalase, paraoxonase-1 higher [186]. The high expression of PON-1 in the liver of older people is important, since this population makes use of large amount of drugs which are metabolized by the liver, and the damage caused by the injury of the hepatic tissue due to the inflammation and oxidative stress can lead to hepatocellular carcinoma [187]. A decrease in PON-1 protein in patients with hepatocellular carcinoma was associated with tumor recurrence, invasiveness, metastasis, as well as with the alterations in cell cycle, DNA replication, gap junctions, and p53 downstream pathways [188]. In patients with colorectal cancer the serum activities of PON1 (paraoxonase and arylesterase) were low in patients with QQ and MM genotypes [189]. In Chinese women the heterozygous and homozygous MM genotypes, and the M alleles, were associated with an increased risk of breast cancer. In addition, the M allele was associated with postmenopausal status and increased nodal involvement [190].

In recent years, overexpression of PON2 and PON3 has been observed in cancer cells and it has been proposed that both enzymes may be involved in tumor survival and resistance to stress [25,75]. Due to its antioxidant effect, PON-2 reduces cellular oxidative damage and influences redox signaling, which promotes cellular survival. Elevated PON-2 levels may stabilize tumor cells by enhancing cellular stress resistance, attenuating mitochondrial ROS-mediated apoptosis [76,191]. Wnt/β-catenin signaling regulates key cellular functions including proliferation, differentiation, migration, genetic stability, apoptosis, and stem cell renewal, as well as maintenance of cancer stem cells, metastasis and immune control [192,193]. In vitro studies have shown that the expression of the PON-2 protein regulates the Wnt/GSK3β/β-catenin pathway in multiple cell lines (K562, SCC-4, PCI-13); besides, a correlation between PON2 and β-catenin expression was observed in *ex-vivo* tumor tissue of oral squamous cell cancer (OSCC). Higher PON-2 expression in OSCC has been associated with tumor relapse, regardless the type of treatment [194]. In addition, a bioinformatic analysis of RNA and DNA sequencing data obtained from tumor samples taken from more than 10.000 patients with 31 different types of cancer indicated that a high level of PON-2 expression correlates with a worst prognosis in patients with multiple types of solid tumors. Moreover, PON-2 located in the nuclear envelope and endoplasmic reticulum, could protect cancer cells against unfavorable environmental conditions and against chemotherapy. Patients with glioblastoma, low grade glioma, liver hepatocellular carcinoma and acute myeloid leukemia, exhibiting higher expression of PON-2, had poor survival when compared with patients with lower PON-2 expression [195].

PON-3 overexpression reduces cytochrome c release and cardiolipin peroxidation, leds to loss in mitochondrial integrity, enhancing cell death resistance in cancers cells by regulating c- MAPK signaling pathway [196]. The mitogen-activated protein kinases (MAPKs) in mammals include c-Jun NH2-terminal kinase (JNK), p38 MAPK, and extracellular signal-regulated kinase (ERK). MAPK signaling pathways contribute to cancer development through the stimulation of cell proliferation, metastasis and drug-resistence [197]. In addition, DNA methylation in PON-3 gene has been associated with both, the recurrence and the resistence to 5-fluorouracil during the treatement in colorectal cancer patients [198], as well as prostate cancer [199].

## 9. Paraoxanase in Healthy Old People

Very few studies have been performed on healthy old people. In a study conducted by Cakatay et al., [200] the paraoxonase and arylesterase activities of PON-1 were investigated in healthy subjects: young (22–45 years), middle-aged (46–65 years) and elderly (66–89 years-old). Paraoxonase and arylesterase activities were reduced in middle-aged and elderly when compared to young people. The decreased activities correlated negatively with oxLDL increase; with the increased oxidation of hydrophobic amino acid residues and hydroxy and hydroperoxy derivatives; and with the increased protein carbonylation and protein oxidation products. However, in the Turkish population, the PON-1 activity was no different between young and old people [201]. A meta-analysis consisting of 5962 subjects, 2795 young (<65 years of age) and 3167 old subjects (>65 years of age), described that subjects carrying RR and QR genotypes (R+ carriers) were favoured in reaching extreme ages, since centenarian and nonagenarian individuals did not have and/or delay the onset of major age-related diseases [202]. In addition, the R allele was related to the increase in PON-1 activity, and the presence of the R allele was associated with longevity in healthy Italian octo/nonagenarians/centenarians [203].

Diet and dietary supplements effects on paraoxonases in the elderly are even less studied. In the frailty syndrome, the PON-1 activity showed no difference when frail lean and frail obese healthy individuals were compared [204]. Moreover, no difference in PON-1 activity was observed by dietary supplementation in the elderly with this syndrome [205].

The Mediterranean diet uses foods such as fruit and vegetables, olive oil, and nuts which are rich in nutrients with important antioxidant properties. Several epidemiological studies suggest that a diet rich in natural antioxidants is associated with protective effects against major diseases present in elderly, especially hypertension, insulin resistance, metabolic syndrome, cardiovascular disease [206]. Extra-virgin olive oil, one of the main sources of fat in this diet, has been effective in increasing the activity of PON-1 [207]. This effect was attributed to the low intake of saturated fatty acids, and to the oleic acid enrichment of the phospholipids in HDL, favoring the increase of the hepatic mRNA of PON-1 and its activity [207]. Intake of extra-virgin olive oil by the elderly over a 12-week period was shown to significantly increase plasma PON-1 concentration and the anti-inflammatory activity of HDL and PON-1 [208]. Interestingly, argan oil has been described to improve aging-related disorders, acting in inflammatory states, and is currently emerging as having antiaging properties [209]. It has been observed that the intake of virgin argan oil for three weeks increased PON-1 paraoxonase activity in up to 31.5% and the PON-1 arylesterase activity up to 45% [210].

## 10. Conclusions and Perspectives

PON-1, -2 and -3 are potent antioxidant enzymes still relatively unknown in their physiological and pathophysiological aspects in advanced age and age-related diseases. Several studies describe contradictory results. Although they are described to be involved in a number of processes, such as oxidative stress, inflammation-related diseases, atherosclerosis and cardiovascular diseases, cancer, neurodegenerative diseases, infectious diseases, to name a few, robust studies are still required to clarify the clinical relevance of PONs.

Significantly more work is needed to fully elucidate PONs physiological functions as well as understand their modulation and their implications for human health. Studies at the cellular level in a variety of tissues, where PONs could locally confer protection from oxidative damage should be performed. Their distinct substrate specificity and localization point out that they may have different functions in the human body. In addition, future studies will have to increasingly focus on PON2 and PON3. Both these enzymes have shown significant antioxidative potential. PON2 is expressed in nearly all human tissues, and thus may be implicated in many other diseases.

A deeper understanding of PON functions and modulators would pave the way for clinical and nutritional interventions, including the development of specific pharmacological agents targeting PONs, dietary modifications and healthy lifestyle (consumption of antioxidants, eating “good” fats or exercising).

Longevity is a complex trait that presumably depends on different combinations of genes, environment and chance, all of which vary quantitatively and qualitatively. More and larger studies are needed to generate data with enough statistical power across all the considered PON genotypes. With these data it may be possible to establish the role and effect of PON polymorphisms in healthy aging across the sexes, in all human populations, and in age-related diseases.
